# Foliar application of fungicide-opera alleviates negative impact of water stress in soybean plants

**DOI:** 10.1016/j.sjbs.2021.02.079

**Published:** 2021-03-06

**Authors:** Mansi Kanungo, K.N. Guruprasad, Sunita Kataria, Gani Asa Dudin, Mohammed Nasser Alyemeni, Parvaiz Ahmad

**Affiliations:** a Department of Biosciences, Christian Eminent College, DAVV, Indore, M.P, India; b Shri Vaishnav Institute of Science, Shri Vaishnav Vidyapeeth Vishwavidyalaya, Indore, M.P, India; c School of Biochemistry, DAVV, Khandwa Road, Indore, M.P, India; d Botany and Microbiology Department, College of Science, King Saud University, Riyadh, Saudi Arabia

**Keywords:** Biomass, Chlorophyll fluorescence, Crop yield, Opera, Photosynthesis

## Abstract

The modulatory effect of opera was investigated on the physiological and morphological aspects in soybean thriving in water stress environment. The data procured from current investigation indicated that water stress significantly declined the plant growth, leaf area in addition to photosynthetic efficiency, nitrate reductase activity and crop yield at various stages of growth such as vegetative (VS), flowering (FS) and pod filling stage (PFS). However, foliar application of opera (0.15%) was effective to enhance the the leaf area (42%), rate of photosynthesis (194%), and nitrate reductase activity (68%) at FS stage while the maximum enhancement in biomass accumulation (92%) and yield (119%) was observed at PFS stage as compared to their control plants. The opera is applied as foliar spray in field experiments to augment the assimilation of nitrogen and carbon in soybean which contributes to increased crop development and productivity under water stress conditions.

## 1 Introduction

There is an extensive assortment of abiotic stresses including heavy metal, drought, salinity that has detrimental effect on plant productivity, plant development and yield efficiency (Kataria and Verma, 2018). Drought stress is the most significant and imperative abiotic stress that significantly affects the crop productivity (Burgess and Huang, 2016, Khan et al., 2018). Drought induces several physiological dysfunctions like instant leaf senescence, prematurity and increased decline in yield of soybean because of decreased seed filing (Manavalan et al., 2009, Bellaloui et al., 2012). The decreased rate of photosynthesis and instant leaf senescence hampers the flow of assimilates accessible to seed that finally determine the seed size in soybean (Wijewardana et al., 2018, Wijewardana et al., 2019).

Drought stress has severe impact on the morphological, biochemical and gene level pathways in plants caused to reduced development, stomata shutting with concomitant decline in transpiration, lower chlorophyll content and impaired photosynthetic rate, declined stomatal conductance and irregular alterations in protein pattern to combat the osmotic variations in their tissues (Makbul et al., 2011, Kosar et al., 2020, Raja et al., 2020, Kaya et al., 2020, Farooq et al., 2020). Even if there are numerous strategies to mitigate the drought stress, the consequences of drought stresses are multiple and interconnected. To surmount these physiological impairments, there are varied options for instance the progress towards the development of tolerant gene lines, seed augmented technologies, employing anti-transpirants, alcohols and particular group of fungicides. Strobilurin is an imperative type of fungicide that originates from *Stroblilurus tenacellus,* the mushroom fungus that is causal agent of wood rotting (Vincelli, 2002). This natural fungicide is anticipated as protectant for fungi against varied microbes in the wood decomposition. The economic feasibility of the strobilurin aggravated scientists to synthesize the man-made strobilurins by chemically modulating the compound to be photostable (Vincelli, 2002).

Opera is synthesized by the German based company BASF Ltd., that is basically suspo-emulsion of two active ingredients 133 g/l (12.5% w/w) pyraclostrobin plus 50 g/litre (4.7% w/w) epoxiconazole derived from strobulirin and triazole family of fungicides (Amin and Jampala, 2018). Earlier research reports have anticipated that employing the fungicides like stobulirin improved growth and productivity of wheat and barley crops even in the absence of pathogens and alter the physiology of crop plants. Majority of the food proteins are derived from the grain legumes as evident in the several regions of the world, legume seeds constitute the distinct protein in the diet. Among all the leguminous crops Soybean is important crop in Indian climatic conditions owing to its immense protein content and elevated percentage of oil (Ahmed et al., 2010) which are main determinants in the seed quality as per the economy (László and RISSAC-HAS, 2010). This incites the demand for soybean productivity worldwide (Brown et al., 2005). However, drought induces the significant decline in the soybean yield (Sadeghipour and Abbasi, 2012).

Consequently, the prime aim of the current investigation is to evaluate the potential impact of water stress particularly on nitrogen and carbon metabolism which finally impacts the crop productivity and the alleviation of adverse effects of water stress through foliar spray of opera on plants. So, our hypothesis was that foliar spray of opera will have beneficial effects on soybean growth and yield and also opera may protect soybean plants from the adverse affects of water stress with reference to carbon and nitrogen metabolism processes; it will greatly help to identify the critical problem of water deficit areas in soybean production.

## 2 Materials and methods

### 2.1 Plant material

Soybean (*Glycine max*), a leguminous crop was used for the current study. The research evaluation was conducted in the field experiments beneath the ambient sunlight on the terrace of the Department of Life Sciences, Indore (22.4°N) for the tenure of 4 months (September to December), 2016. Seeds of soybean (*Glycine max* L. *Merrill* variety: JS-335) were procured from Indian Institute of Soybean Research, Khandwa Road, Indore, Madhya Pradesh, India. Wet soybean seeds were coated with Rhizobium (*Rhizobium japonicum*) culture *via* uniform smattering of aforesaid culture used for all the seeds control as well as opera treated. Ten seeds of similar dimensions were packed in plastic bags (34 X 34 cm) and after germination six plants were kept in each bag. Each bag was loaded with 5 kg mixture of sand, black soil and cow dung manure in proportion of 1:2:1 by volume for all the treatments used. The black soil used was rich in lime, iron, alumina and magnesia. The average temperature ranged from 27 to 30 °C, relative humidity ranged from 55 to75% during the experimental period. Plants were irrigated regularly excluding the bags subjected to drought stress experiments. The experiment was conducted in a randomized block design with three replicates; 3 plastic bags for each treatment (0%, 0.05%, 0.15% and 0.3% opera).

### 2.2 Chemical treatment

Foliar application of Opera (Pyraclostrobin + Epoxiconazole) was applied at a recommended rate of 1.5 L per ha on soybean leaves at 10 and 20 DAE (Days after Emergence).

#### 2.2.1 Concentrations used

Effect of different concentration of opera (0.05%, 0.1%, 0.15%, 0.2% 0.25% and 0.3%) were studied for soybean growth and yield (data not given) in ambient conditions and three concentrations of opera (0.05%, 0.15% and 0.3%; lower, optimum and higher respectively) were recommended for further experiments.

### 2.3 Water stress conditions produced at different growth stages of soybean

To evaluate the impact of opera on soybean under water stress condition, stress was imposed by withholding irrigation in three experimental conditions at different crop growth stage: like vegetative, reproductive, grain filling stages.

**1. Vegetative VS (at 30 DAE):** stress was applied when the plants had four fully expanded leaves.

**2. Reproductive FS (at 40 DAE):** was applied at the beginning of flowering.

**3. Pod filling stage PFS (at 60 DAE):** stress was applied when the seed formation started.

Plants subjected to drought stress for 5 days through impeding the irrigation at 30, 40 and 60 DAE after that these plants were re-watered till its maturity (120DAE). For comparison, one more experimental setup of plants was maintained as control (well watered) where these plants received irrigation throughout the cropping period.

### 2.4 Growth parameters

#### 2.4.1 Leaf area

The area of third trifoliate leaves was estimated by using Leaf Area Meter CI-202 (CID Bio Sciences, NW Camas, WA).

#### 2.4.2 Crop yield and yield attributes

Once the crop reached the maturity at 120 DAE five replicates of three experimental set ups were harvested and yield attributes like 100 seed weight, number of pods, number of seeds and seed weight per plant was estimated. Pods were separated and the total number of pods were recorded. At harvest maturity (120 DAE) the yield parameters of soybean were measured.

### 2.5 Physiological analysis

#### 2.5.1 Photosynthetic pigments

The estimation of chlorophyll content was carried in third trifoliate leaves from which have acquired full expansion after 45 DAE *via* dimethyl sulfoxide (DMSO) method (Hiscox and Israelstam, 1979). Leaves weighing 50 mg were immersed in 5 ml of DMSO overnight at room temperature. The absorbance was taken at 480, 649 and 665 nm using Shimadzu UV/VIS 1601 spectrophotometer (Kyoto, Japan). The Chl a, Chl b, and total chlorophyll concentration was deduced from Wellburn’s equation (Wellburn and Lichtenthaler, 1984).

#### 2.5.2 Fluorescence measurements

The third trifoliate leaves of soybean plants were subjected to Handy PEA (Plant Efficiency Analyzer) fluorimeter, Hansatech Instruments, Pentney, England) for estimating the transient chlorophyll a fluorescence. Samples were exposed to homogenous illumination via six light emitting diodes targeted on 4 mm diameter mark on the leaf surface that induce the transients by red light (peak at 650 nm) of 600 Wm^2^ (3,200 μE m^−2^ s^−1^). The observations were taken for 1 s with 12-bit resolutions and each value was acquired after every 1 ms afterwards (Strasser et al., 1995). The values were acquired at 25 ± 1 °C. The Polyphasic fluorescence rise in kinetics (O–J–I–P phase) was displayed when Chl a fluorescent transient was plotted against logarithmic scale. The intensity at O phase was designated as fluorescence intensity at 20 µs designated as Fo where all the reaction centres are opened, J phase determines the fluorescence intensity at 2 ms while as 30 ms was represented as I phase and maximum fluorescence (Fm) was marked at P phase (Fp = Fm) because the excitation intensity is too elevated to guarantee the termination of all the reaction centres of the photosystem II (PSII) (Kalaji et al., 2016, Kalaji et al., 2018). Using JIP test, OJIP transient was evaluated and subsequent attributes was estimated and maximum quantum yield of primary photochemistry (Fv/Fm) equivalent to the efficiency was determined following the methodology of Baghel et al. (2016). OJIP transients were normalized to Fo. JIP test parameters were measured such as Fv/Fo, a value that is proportional to the activity of water splitting complex on donor side of PSII or the primary photochemistry of PSII; and PI/ABS, performance index at absorption basis that reflecting the performance of the overall energy flow. The Biolyzer HP3 software (Bioenergetics Laboratory) was used for calculation of measured J-I-P test parameters.

#### 2.5.3 IRGA analysis

Gas exchange parameters such as rate of internal CO_2_ concentration (*Ci*) (μmol CO_2_ mol^−1^), photosynthesis (*Pn*) (μmol CO_2_ m^−2^ s^−1^), stomatal conductance (*gs*) (mmol H_2_O m^−2^ s^−1^) and transpiration rate (*Tr*) (mmol H_2_O m^−2^ s^−1^) were measured in the leaves of each treatment (entirely open third leaf from the top at 45 DAE) by means of a HandyInfra Red Gas Analyzer (Li-6200, LI-COR Inc., Lincoln, NE).

Photosynthetic photon flux density (PPFD) was estimated in entire plant thriving under field environments with ambient climatic conditions like standard CO_2_ concentrations and temperature on sunny days. CO_2_ concentration (350–380 ppm), photosynthetic photon flux density (PPFD) (1,300–1,600 μmol m^−2^ s^−1^), and air flow (500 μmol s^−1^) was recorded on sunny day between 11.00 and 12.00. All these attributes were estimated by IRGA were observed thrice for every sample.

#### 2.5.4 Determination of nitrate reductase (NR) activity [E.C. 1.6.6.1]

Leaves weighing 100 mg were harvested, chopped and subjected to incubation for 2 hrs at 30 °C in a homogenate including phosphate buffer (0.1 M), potassium nitrate (0.2 M) and isopropanol (25%) and nitrate content was estimated following procedures of Jaworski (1971). The absorbance of the nitrate was recorded at 540 nm and the units of NR were measured as nM NO_2_ g^−1^ FW h^−1^.

### 2.6 Statistical analysis

All the observations recorded are calculated from experiments that were repeated thrice in five plants for each replicate. Data values are presented as means ± S.E.M and were investigated *via* Analysis of Variance (ANOVA) tagged on *via* posthoc employing Graph pad software, La Jolla, CA, USA.

## 3 Results

### 3.1 Leaf area

**Fig. 1:**
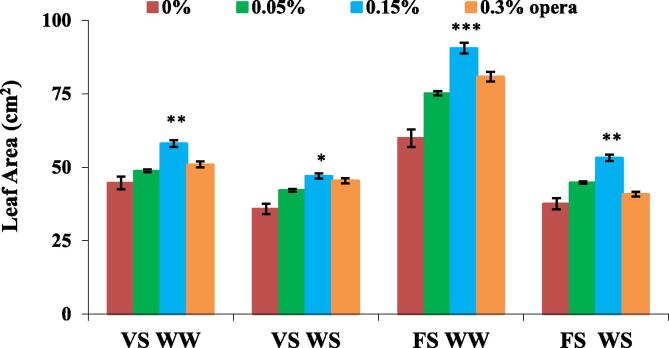
Impact of foliar application of opera on area of third trifoliate leaves of soybean in well-watered and water stress conditions given at VS and FS stages. The vertical bar indicates ± SE for mean ***, ** and * indicate significance at P < 0.001, 0.01 and 0.05 respectively, compared to their control.

Leaf area decresed in both vegetative and flowering stage under water stressed soybean plants. However, plants subjected to 0.15%opera displayed significant enhancement in leaf area at both vegetative and flowering stage under water stress conditions respectively by 31% and 42%in comparision to the control (Fig. 1).

### 3.2 Chlorophyll content

**Fig. 2:**
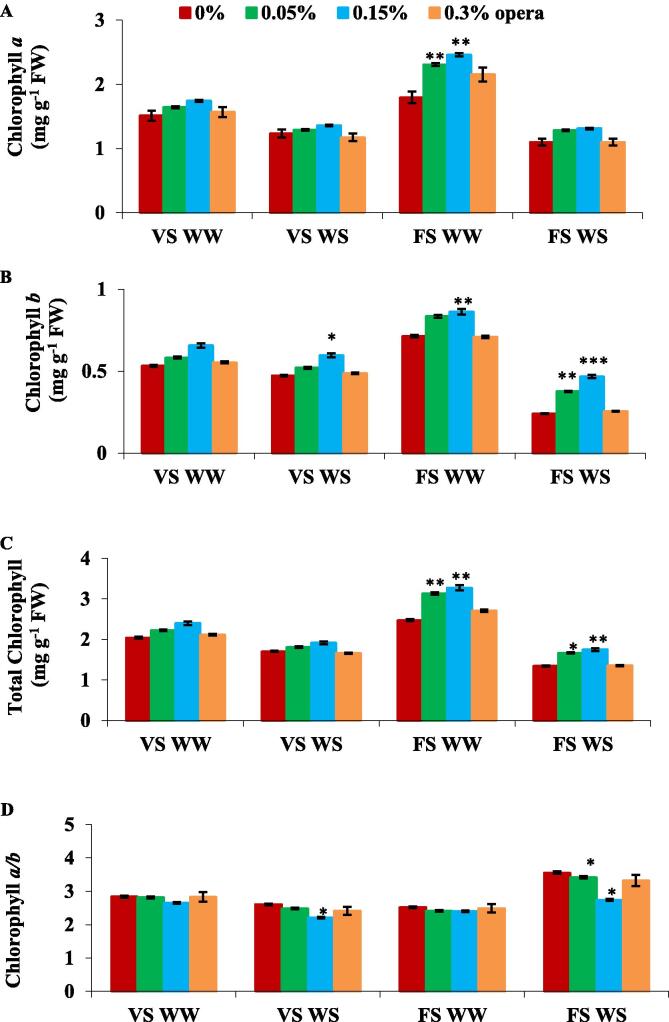
Impact of foliar application of opera on chlloropyll *a* (A), Chl*b* (B), total Chl (C) content and Chl*a*/Chl*b* ratio (D) of soybean in well-watered and water stress conditions given at VS and FS stages. The vertical bar indicates ± SE for mean ***, ** and * indicate significance at P < 0.001, 0.01 and 0.05 respectively, compared to their control.

Water stress lead to the decline in uniformly fraction in chlorophyll *a* and *b* in third trifoliate stage of soybean plants in comparison to well irrigated plants but under water stress conditions considerable increase in chlorophyll content was displayed in plants exposed to 0.15% opera (Fig. 2A,B,C). In soybean 0.15% opera showed enhancement in total chlorophyll content by 12% and 30%, at VS and FS correspondingly, in contrast to control plants (Fig. 2C).

The significant rise in total chlorophyll concentration was as a result of higher increment in chlorophyll *b* (26% and 93% at VS and FS respectively) than chlorophyll *a* (10%, and 19% at VS and FS respectively) (Fig. 2A-C). Because of this, there was significant decline in chlorophyll *a/b* ratio following application of 0.15% opera (15% and 23% reduction at VS and FS respectively) (Fig. 2D).

### 3.3 Chlorophyll *a* fluorescence

**Fig. 3:**
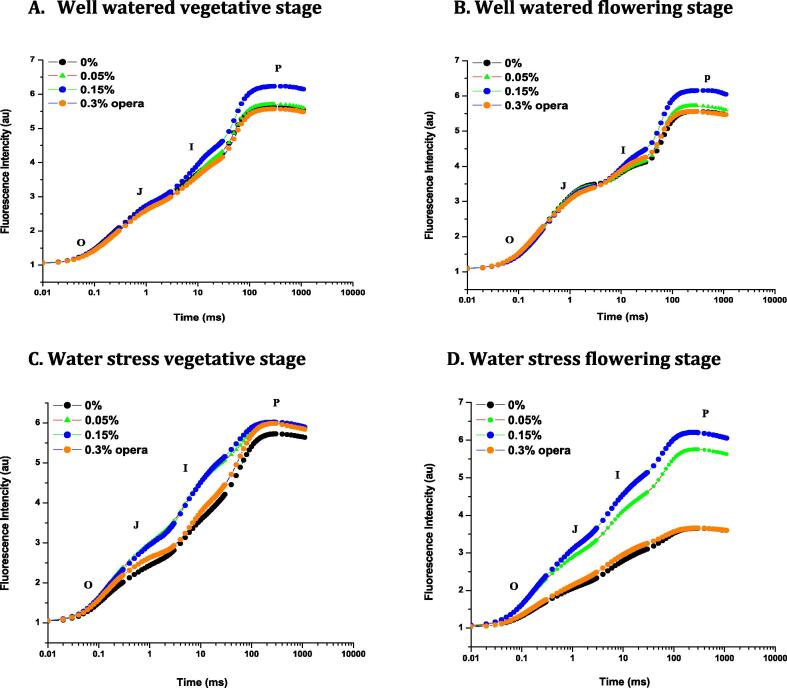
Impact of foliar application of opera on polyphasic chlorophyll *a* fluorescence transient curve in leaves of soybean in well-watered and water stress conditions given at VS and FS stages. The vertical bar indicates ± SE for mean ***, ** and * indicate significance at P < 0.001, 0.01 and 0.05 respectively, compared to their control.

The effect of opera was more pronounced on the photochemical efficiency of PSII under water stress as observed *via* polyphasic chlorophyll *a* fluorescence transient. Separation of OJIP phase was displayed on logarithmic time scale as duration of fluorescence yield in soybean leaves under dark was plotted for both watered and stressed soybean plants (Fig. 3A-D). The intensity of fluorescence was drastically decreased at J-I-P phase in control ones when water stress was given on flowering stage but it was enormously increased after the application of 0.15% opera (Fig. 3D).

**Fig. 4:**
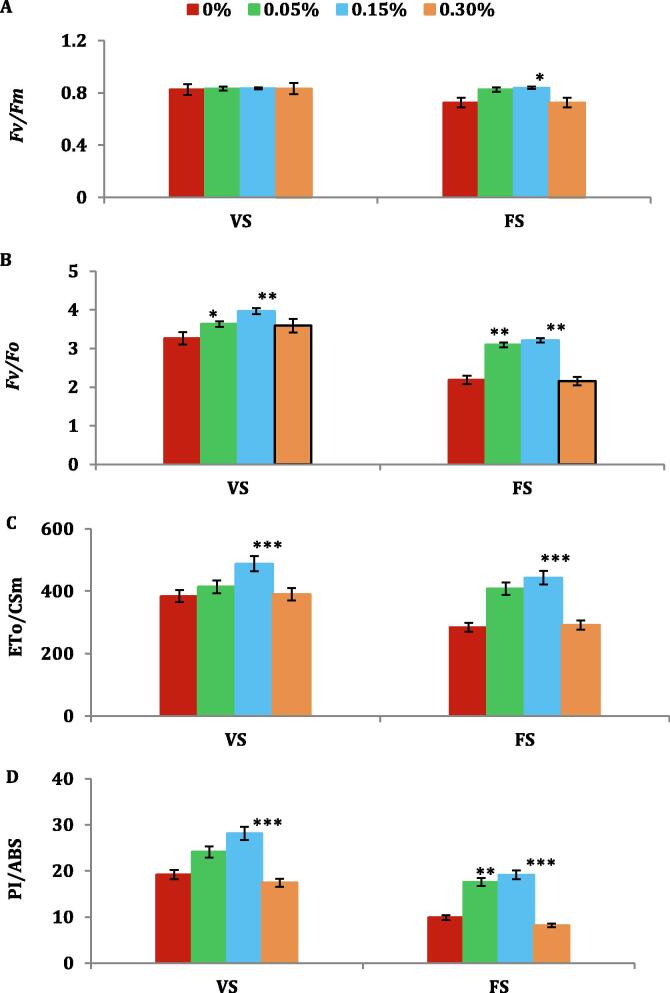
Impact of opera on soybean OJIP parameters of soybean leaves under water stress conditions given at VS and FS stages; *Fv/Fm* (A), *Fv/Fo* (B), ETo/CSm (C), and PI/ABS (D). The vertical bar indicates ± SE for mean ***, ** and * indicate significance at P < 0.001, 0.01 and 0.05, respectively as compared to control.

The highest quantum yield of primary photochemistry (Fv/Fm) exhibited meager/no significant disparity between opera supplemented plants and control plants (Fig. 4A). Water stressed leaves of control plants exhibited more obvious decline in electron transport per leaf CS (ETo/CSm) while 0.15% opera lead to significant enhancement of 27% and 56% at VS and FS correspondingly compared to untreated control plants thriving under water stress conditions (Fig. 4C). The sample vitality is most sensitive attribute to be estimated *via* the JIP test which is the performance index (PI_ABS_). PI_ABS_ displayed decline in water stress of leaves in untreated plants. PI_ABS_ was considerably increased by 0.15% of opera treatment under water stress conditions. Though, PI_ABS_ was increased by 46% and 93% opera concentration of opera at VS and FS respectively in comparison to untreated plants grown in control condition (Fig. 4D).

### 3.4 CO_2_ fixation

**Fig. 5:**
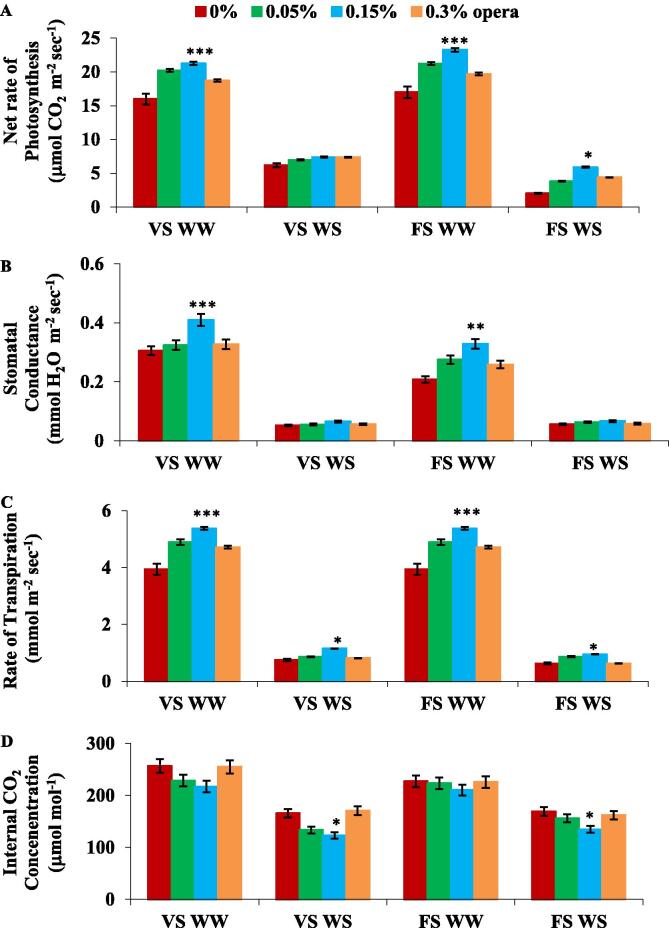
Impact of foliar application of opera on gas exchange parameters photosynthetic rate (A), stomatal conductance (B), rate of transpiration (C) and internal CO_2_ concentrations (D) of soybean leaves in well-watered and water stress conditions given at VS and FS stages. The vertical bar indicates ± SE for mean ***, ** and * indicate significance at P < 0.001, 0.01 and 0.05 respectively, compared to their control.

IRGA was employed for assessing the physiological attributes that presents thorough confirmation for the sharp increase in CO_2_ fixation. For this experiment, third trifoliate leaves were excised from intact plants and subjected to IRGA to acquire the data pertaining to the inter-cellular CO_2_ concentration, stomatal conductance plus net rate of photosynthesis and transpiration rate. All these parameters were recorded and display decline in water stressed soybean plants (Fig. 5 A, B, C, D). In soybean plants, opera treatment (0.15%) resulted in marked rise in stomatal conductance plus net rate of photosynthesis and transpiration rate under water stress in addition to irrigated soybean plants in comparison to untreated control plants, apart from that inter-cellular CO_2_ content declined imprecisely. An increase of 20% and 94% in net photosynthesis was recorded (at VS and FS correspondingly) in seedlings exposed to 0.15% opera in comparison to controls under water stress environment (Fig. 5A). Enhancement of 24% and 18% in stomatal conductance and 54% and 51% enhancement in transpiration rate at VS and FS were obtained after 0.15% opera (Fig. 5B,C). Opera treatment resulted in 26% and 20% decline in inter-cellular concentration of CO_2_ at VS and FS in that order, in comparison to untreated control under water stress condition (Fig. 5D).

### 3.5 Nitrate reductase (NR) activity

**Fig. 6:**
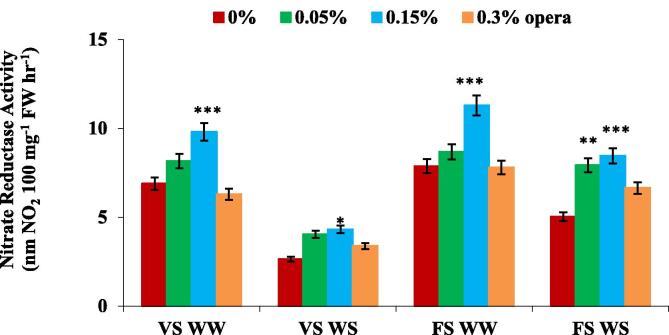
Impact of foliar application of opera on nitrate reductase activity in leaves of soybean in well-watered and water stress conditions given at VS and FS stages. The vertical bar indicates ± SE for mean ***, ** and * indicate significance at P < 0.001, 0.01 and 0.05 respectively, compared to their control.

Opera treatment at 0.15% resulted in impaired nitrogen metabolism; in this treatment significant enhancement was found in activity of nitrate reductase under water stress as well as well watered conditions. NR activity declined in water deficient environment applied at varied crop growth stages in comparison to non-stressed plants. But after the foliar spray of opera activity was significantly enhanced under water stress as well as well watered condition. NR activity was enhanced by 63% and 68% by opera treatment (0.15%) at VS and FS respectively under water stress condition (Fig. 6).

### 3.6 Biomass and yield

**Fig. 7:**
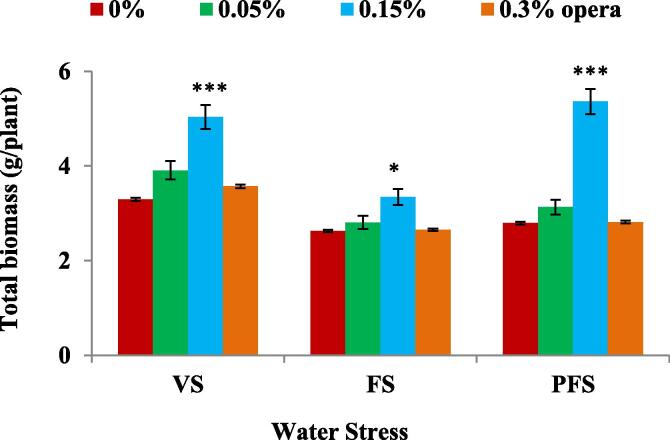
Impact of foliar application of opera on total biomass of soybean in well-watered and water stress conditions given at different growth stages (VS, FS and PFS) of soybean. The vertical bar indicates ± SE for mean ***, ** and * indicate significance at P < 0.001, 0.01 and 0.05 respectively, compared to their control.

**Fig. 8:**
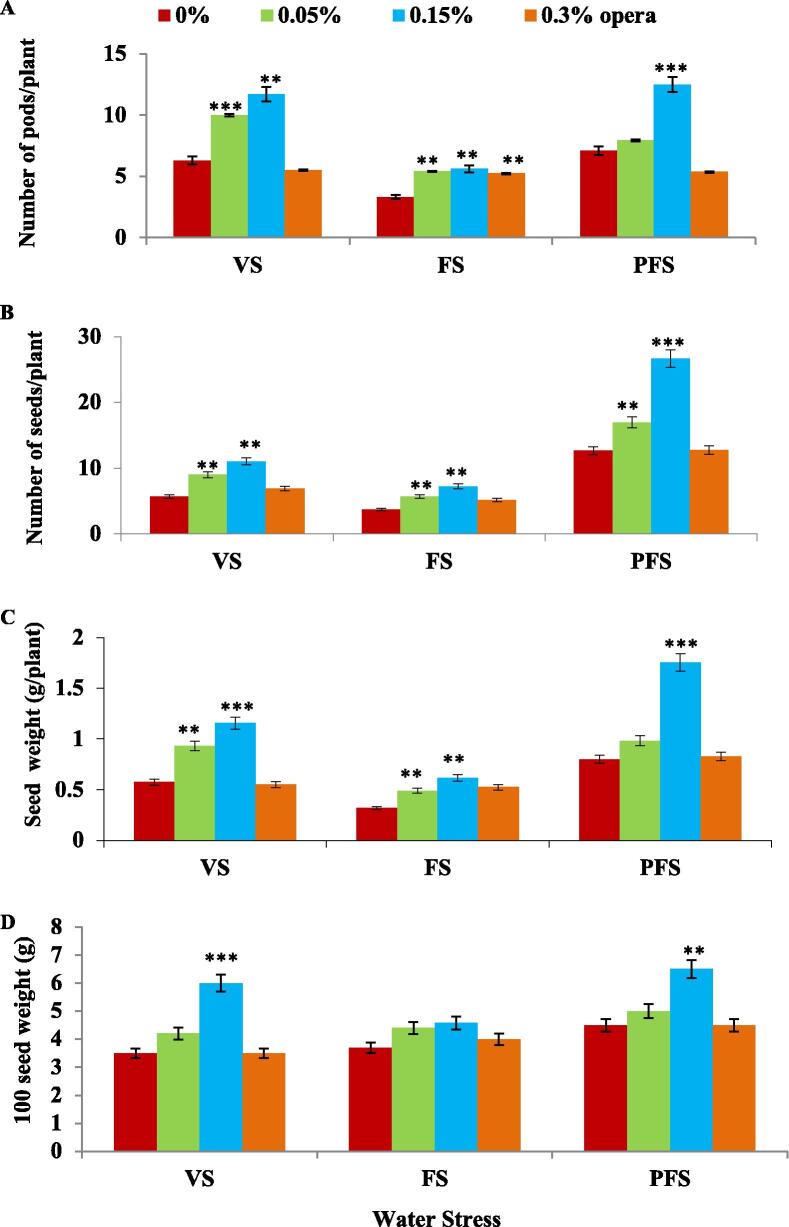
Impact of foliar application of strobilurin opera on soybean yield and its attributes such as number of pods/plant (A), number of seeds/plant (B), seed weight g/plant (C) and 100 seed weight (g) (D) in well-watered and water stress conditions given at different growth stages (VS, FS and PFS) of soybean. The vertical bar indicates ± SE for mean *** and ** indicate significance at P < 0.001, 0.01 and 0.05 respectively, compared to their control.

**Fig. 9:**
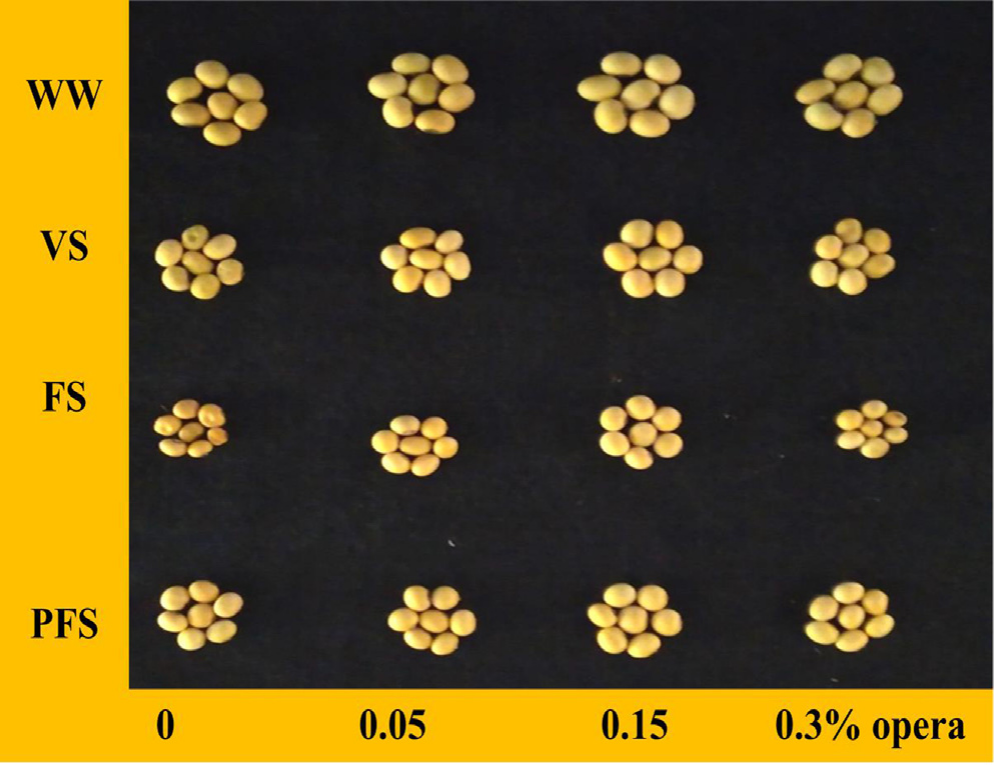
Impact of foliar application of opera on size of soybean seeds in well-watered and water stress conditions given at different growth stages (VS, FS and PFS) of soybean. The vertical bar indicates ± SE for mean ***, ** and * indicate significance at P < 0.001, 0.01 and 0.05 respectively, compared to their control.

Soybean plants supplemented with 0.15% opera displayed increased biomass under water stress compared to control plants sans opera application as observed at all the three stages of water stress given to the plants (vegetative, flowering and seed filling) i.e. (52%, 27% and 92% respectively) (Fig. 7). Water stress lead to decline in the overall yield attributes in comparison to irrigated plants (Fig. 8, Fig. 9). Soybean plants supplemented with 0.15% opera exhibited significant increase in number of pods/seeds per plant, and seed weight per plant in comparison to control water stressed plants alone. Maximum rise in number of pods was recorded as 86%, 70% and 76% in comparison to untreated controls at VS, FS and PFS respectively (Fig. 8A).

Increase in the number of seeds per plants on 0.15% opera application was 94%, 96%, and 111% in comparison to their untreated controls at VS, FS, and PFS, correspondingly (Fig. 8B). Maximum increase in seed weight was observed as 101%, 92%, and 119%, respectively, at VS, FS, and PFS by 0.15% opera treatment in comparison to their untreated controls (Fig. 8C). Correspondingly, 100 seed weight was enhanced over 71%, 23% and 44% (at VS, FS and PFS respectively) after the application of opera in comparison to their respective water stress only plants (Fig. 8D). Fig. 9 illustrates the increased size of soybean seeds after the foliar application of opera in well-watered and water stress conditions.

## 4 Discussion

Drought stress caused restrictions of growth and productivity of plants through noteworthy alternation at the cellular, physiological, molecular and biochemical levels (Burgess and Huang, 2016). The data procured from all the experiments displayed upgraded advancement by opera at 0.15% as reported in varied growth attributes along with the positive impact on the overall carbon and nitrogen metabolism in both water stressed and well irrigated soybean seedlings. Opera at 0.15% resulted in the modulatory effect on all growth aspects for instance leaf area and biomass accumulation as recorded at various developmental stages of soybean. This enhancement in the overall growth aspects can be attributed to the positive impact of the 0.15% opera on the carbon and nitrogen metabolism. Slusarenko et al. (2012) reported the inhibitory implication of foliar sprayed pyraclostrobin on the ethylene biosynthesis in wheat shoot thriving under short term drought stress. Percival and Noviss (2008) also documented initiation of drought tolerance in horse chestnut trees (*Aesculus hippocastanum*) following the foliar spray of triazole (Epoxiconazole, propiconazole, penconazole or paclobutrazol).

Soybean seedlings treated with 0.15% Opera exhibited higher water potential than the control seedlings. Davis et al. (1988) reported comparable observations with triazole in plants thriving in drought stress environment. The modulatory effect of strobilurins on drought stress has been attributed to its competence for impeding the water uptake that leads to restructuring of soil dehydration (Inagaki et al., 2009) which adds to the grain yield in few wheat genotypes thriving under water stress. Current study concludes that the application of opera had an immense effect on assimilatory surface area and its allied characters. Leaf area is a significant factor of photosynthetic capacity in the plants. The application of opera increased the leaf area in comparison to control and more increase was reported at 0.15% opera concentration in both well irrigated and water deficit conditions. Since, leaf senescence is one of the most obvious constraints during peak grain filling period and application of strobilurin arrested the chlorophyll degradation which ultimately helped in delaying senescence. Percival and Noviss (2008) has also reported high total chlorophyll/ proline content and elevated photosynthetic rates than untreated seedlings following three week drought. Our results also showed the enhancement in photosynthetic pigments like chlorophyll *a, b* and total chlorophyll after the application of 0.15% opera which decreased in untreated control plants under water stress conditions. Petit et al. (2008) has demonstrated hermetic response in physiological processes like photosynthesis following the foliar spray of fludioxonil, as fungicide induced the variation in photosynthetic activity of plants as its application induces decline in the photosynthetic rate in grapevine. In current research study, 0.15% opera treated soybean seedlings display elevated fluorescence yield at the J–I–P phase in polyphasic chlorophyll *a* fluorescence (OJIP) transients in both well irrigated plus water stressed soybean seedlings.

Application of opera leads to the higher ascent in fluorescence as it causes the instant decline of electron acceptors in photosynthetic pathway downstream of PSII particularly plastoquinone and Q_A_ (Maxwell and Johnson, 2000). Current research investigation exhibited the high quantum efficiency in PSII as estimated from Fv/Fm and competence of the water splitting complex on the donor side of PSII as inferred from Fv/Fo declined in seedlings thriving under water stress. However, Fv/Fo was elevated with 0.15% opera application in both water stresses (VS and FS) in comparison to control soybean seedlings. Water stress reduced electron transport per leaf CS (ET_0_/CSm) which was increased by 27% and 56% in soybean following opera application at rate of 0.15% in both water stressed seedlings at vegetative and flowering stages in comparison to untreated seedlings.

Opera application at rate of 0.15% leads to higher performance index (PI_ABS_) over the untreated controls in non-stressed seedlings. The maximum decrease in performance index (PI_ABS_) under water stress was found at flowering stage. PI_ABS_ was enhanced by 46% and 93% in soybean by opera respectively, at vegetative and flowering stage in comparison to untreated plants. This attribute covers the fluorescent variations linked with fluctuations in the antenna conformation and energy variations. The improvement in the physiological attributes for instance gas exchange estimations was verified by elevated rates of photosynthetic rates of strobilurin treated wheat seedlings growing in the open field trials which led to yield benefitted up to 9%. Our result also showed the similar effect of strobilurin opera on gas exchange parameters under stressed and non stressed conditions.

Nitrate reductase catalyzes the reaction that reduces the nitrates to nitrites which is actually primary step in the nitrogen assimilation. Glaab and Kaiser (1999) has elaborated the effect of strobilurins on nitrogen dynamics as they impact the nitrate reductase activity directly. In current investigation, nitrate reductase activity increased in soybean leaves following 0.15% opera application in both well irrigated as well as water stressed seedlings. Bayles and Hilton (2000) demonstrated a normal increase in yield about 0.75 and 0.34 mg ha^−1^in wheat and barley seedlings in United Kingdom because of high nitrate assimilation. Similar results were acquired with corn, wheat and soybean (Henry et al., 2011, Nelson and Meinhardt, 2011, Hill et al., 2013). Our results also showed decrease in total biomass accumulation under water stress at all stages of stress as compared to non stress or well watered plants of soybean crop. Soybean seedlings on 0.15% opera application exhibited significant enhancement in biomass at all stages of water stress. Opera enhanced the NR activity in our study may be due to higher nitrite levels that can improve the growth of plants and also higher NR activity may leads to increase nitric oxide production in opera treated plants as compared to control. Strobilurins also activates the NR activity in plants due to activation of the alternative oxidative pathways of mitochondrial respiration, which decrease the cellular level of ATP while increase the H + in cytosol (Glaab and Kaiser, 1999). Recently, it was found that NO plays an important role in response of plants to abiotic stresses (Nabi et al., 2019, Kataria et al., 2020). However, we found that water stress caused a considerable reduction in photosynthesis rate, chlorophyll content and chlorophyll fluorescence which is more likely to be responsible for the reduction in growth and biomass accumulation. The performance index are related to the productivity of photosynthetic metabolites and in the present study higher PI(ABS) of plants after foliar spray of opera to soybean plants may contributes to higher efficiency of light harvesting and consequently increased biomass in treated plants.

Data obtained indicates that the soybean plants exposed to 0.15% opera had more pods and seeds and the maximum seed productivity. Thus this is the first study in soybean plants foliar sprayed with 0.15% opera, results in improved crop yield due to increased carbon and nitrogen metabolism. Therefore, it had been concluded that 0.15% opera application rearranged the carbon and nitrogen metabolism which augments the crop yield thriving under both irrigated and water deficit conditions. The pod setting and flowering stages are most susceptible phases in the chickpea and soybean (Liu et al., 2004, Nayyar et al., 2006). Results of greater yield reduction under water stress at flowering stage were also observed in our results. Plants of soybean showed significant enhancement 96% in seed yield under water stress after the application of 0.15% opera compared to untreated control plants under water stress given at flowering stage. Increased productivity following application of strobilurin is ascribed to the non-fungicidal physiological alteration as observed in wheat (*Triticum aestivum* L*.*) and barley (*Hordeum vulgare* L.) (Bayles and Hilton, 2000). Assembling all the recorded observations, it can be anticipated that the impact of opera can results in improved enterprise of soybean plant development and productivity under water stress conditions. Opera concentration at 0.15% can be employed for augmenting the growth and yield attributes by curtailing the stress induced deleterious effects in diverse crops.

## 5 Conclusion

All the observations recorded in the experiments concluded that the opera application at 0.15% as foliar spray modulates the crop productivity in terms of elevated carbon and nitrogen metabolism. Higher carbon and nitrogen metabolism manifest in terms of marked rise in the photosynthetic efficiency and high NR activity. Opera helped in chlorophyll retention of plants at later stages even under water stress conditions also, which lead to enhanced fixation of CO_2_ and resulted in the better growth and yield. Opera treatment induced nitrate reductase activity in the soybean leaves, plays an important role in pod filling under water stress and well-watered conditions. Therefore, Opera can be recommended to farmers for augmenting the crop growth and productivity which could yield higher production of commercially important crops albeit stress, arid and harsh environments.

**Declarations**

Ethics approval: Not Applicable

Consent to participate: All authors consent to participate in this manuscript

Consent for publication: All authors consent to publish this manuscript in Saudi journal of Biological Science

Availability of data and material: Data will be available on request to corresponding or first author

Code availability: Not Applicable

## Declaration of Competing Interest

The authors declare that they have no known competing financial interests or personal relationships that could have appeared to influence the work reported in this paper.
